# Present and past dynamics of Inughuit resource spaces

**DOI:** 10.1007/s13280-018-1039-6

**Published:** 2018-03-08

**Authors:** Janne Flora, Kasper Lambert Johansen, Bjarne Grønnow, Astrid Oberborbeck Andersen, Anders Mosbech

**Affiliations:** 1 Department of Anthropology, University of Copenhagen, Øster Farimagsgade 5, 1353 Copenhagen K, Denmark; 2 Department of Bioscience, Aarhus University, Frederiksborgvej 399, 4000 Roskilde, Denmark; 3 Arctic Research Centre, Department of Bioscience, Aarhus University, Frederiksborgvej 399, 4000 Roskilde, Denmark; 4 The National Museum of Denmark, Frederiksholms Kanal 12, 1220 Copenhagen K, Denmark; 5 Department of Learning and Philosophy, The Techno-Anthropology Research Group, Kroghstræde 3, Building 4249, 9220 Aalborg Ø, Denmark

**Keywords:** GPS tracking, Hunting, Mobility, Networks, Resource spaces, Seasonality

## Abstract

Information from a collaborative GPS tracking project, *Piniariarneq*, involving 17 occupational hunters from Qaanaaq and Savissivik, Northwest Greenland, is used to explore the resource spaces of hunters in Avanersuaq today. By comparison with historical records from the time of the Thule Trading Station and the decades following its closure, we reveal a marked variability in resource spaces over time. It is argued that the dynamics of resources and resource spaces in Thule are not underlain by animal distribution and migration patterns, or changes in weather and sea ice conditions alone; but also by economic opportunities, human mobility, settlement patterns, particular historical events and trajectories, and not least by economic and political interests developed outside the region.

**Electronic supplementary material:**

The online version of this article (10.1007/s13280-018-1039-6) contains supplementary material, which is available to authorized users.

## Introduction

There is a little disagreement among people in Avanersuaq (Thule area) today that the conditions underpinning hunting have undergone changes in recent years. Some relate to environmental changes, e.g., global warming that causes glaciers to retreat and reduces sea ice, thereby gradually eroding the foundation of dog sledge infrastructure. Others relate to social and economic changes, such as the economic means to purchase fast motor boats needed to utilize the expanding open water season, or embracing Greenland halibut fishery or tourism, which both provide new opportunities for many occupational hunters and the community more generally (Hastrup 2015: 190 f.).

The purpose of this paper is to explore the ways in which occupational hunters in contemporary Avanersuaq utilize their resource spaces. Initially, we provide a brief outline of the role and significance of hunting in Avanersuaq today, arguing that the notion of subsistence is intertwined with cash economy. The core of the paper is structured around data from our collaborative GPS tracking project, *Piniariarneq* (a sub-project under the NOW Project) (Andersen et al. 2017), in which 17 occupational hunters from Qaanaaq and Savissivik documented their hunting trips during 13 months in 2015–2016. These data illustrate a highly dynamic approach to how, when, and where hunters catch and utilize resources; both spatially across the entire region, and temporally through the different seasons of the year. We compare the *Piniariarneq* data with historical records from the early to late twentieth century: from the period of the Thule Trading Station (1910–1953) (Rasmussen 1921; Holtved 1967) and the years that followed until the mid-80s (Gilberg 1971, 1984).^1^ We use this time depth to support our analysis of the multiple factors—social, cultural, technological, and environmental occurring inside and outside the region—that drive the formation and dynamics of resource spaces. Finally, we assess *Piniariarneq* as a tool and method, which allows for different forms and regimes of knowledge to be integrated into spatial planning of the utilization of living resources.

The concept of resource space is not one that is discussed or defined in anthropology, archaeology, or biology. However, it is one we have found particularly relevant and “good to think [with]” (Levi-Strauss 1991 [1962]: 89) in our endeavour to trace long-term dynamics in hunting practices, and resource and landscape use in Avanersuaq; not merely in terms of where and when animals migrate, or humans travel and hunt; but in terms of when, where, how, and why they meet. What we take resource spaces to mean, therefore, requires some initial clarification. The term “resource space” alludes to a geographical area with an abundance of resources. In our case, we focus specifically on living resources. Such an area may sometimes be quite finite (a seabird colony), and sometimes in flux (an ice lead where a group of animals on migration occurs, or the retreating edge of sea ice where hunters pursue marine mammals in spring). Our study of resource spaces has conceptual, methodological, and analytical purposes. The *Piniariarneq* project sought to map resource spaces by trying to ascertain the location and seasonality of resource use according to the hunters’ own GPS registrations. Resource spaces, therefore, necessarily unfolded with the hunters’ movements in the landscape.^2^ Our understanding and employment of resource space, then, is that it emerges from the encounter between animals (living resources) and humans (hunters). They are underpinned spatially, temporally and not least by social practice (cf. Giddens 1979; Ortner 1984). For the purpose of this paper, there cannot be resources or resource spaces without utilization: it takes a human to perceive something as a resource in the first place, and it takes actual harvest of this resource for a resource space to appear.

One thing that emerges quite clearly from the *Piniariarneq* data is that resource spaces are part of larger social processes that in many ways are similar to “events”. Like weddings, revolutions, and national holidays, resource spaces occur at a certain time and place. However, they also incorporate interests and social structures whose trajectories reach far beyond the boundedness of these (e.g., Moran 2005; Kapferer 2015). The meaning, occasion and purpose of say, a revolution, a public speech, or a resource space, is not only situated in its own time and space, but come about and set in motion things and processes beyond themselves. We might say that resource spaces “occur”, rather than simply “are”. They form, move, and shift, and, like events, they are generative, which is to say they allow (or may even have the purpose) of endowing humans with agency to reconfigure their own possibilities and constraints in new ways.

Our main argument is that resource spaces—the spatial and temporal nodes in which resource use occurs—are dynamic events that emerge, disappear, re-emerge (e.g., seasonally), and transform over space and time. We will show how resource spaces are affected by historical trajectories, and that the dynamics of resource spaces are influenced by many interconnected factors such as opportunities available to the hunters; the gradual formation and retreat of sea ice; fluctuations in movement of animals; as well as by settlement patterns, and not least by political and economic interests originating outside the region beyond the resource spaces themselves.

## Hunting in contemporary Avanersuaq

There are just under 800 people living in Avanersuaq today. Most of them (around 650) live in the region’s largest town, Qaanaaq, and the rest live in the three villages Siorapaluk, Qeqertat,^3^ and Savissivik.^4^ The former two are in vicinity to Qaanaaq, while Savissivik is located in the southernmost part of the region, facing Qimusseriarsuaq (Melville Bay). Though everyday life in this part of Greenland carries many similarities with the rest of the country, there are also numerous ways in which Avanersuaq and the people who live there (predominantly Inughuit), set themselves apart. Hunting traditions, tools, and technology differ in some ways from those of other hunting communities in Greenland, and hunters in the region adamantly proclaim that these characteristics are distinctly theirs. Narwhals (*Monodon monoceros*) are notoriously skittish and hunters, therefore, insist on catching them from kayak to avoid disturbance of the animals from the noise produced by a motorized boat. Narwhals should also be harpooned to prevent them from sinking to the ocean floor. Similarly, polar bears (*Ursus maritimus*) are hunted using dogs that have been specially trained for this purpose, and motorized vehicles (snowmobiles, motorboats etc.) are prohibited in certain areas during certain parts of the hunting season.^5^ Furthermore, the catch should be shared according to local prescriptions and methods of sharing.

The seasonal migrations of animals to and from the region has rendered it possible to talk of a hunting calendar (Steensby 1917; Gilberg 1984; Born 1987; Grønnow 2016), alluding to an image of continuity and cyclical predictability in hunting practices and resource use over time. While such an approach is useful for gauging seasonal resource use, it is one, we should caution, that reduces complex human practices to a model, and tends to produce an image of Arctic timelessness, which has been widely critiqued (Fienup-Riordan 1990; Steckley 2008). Despite the many social, economic, and cultural changes that have occurred over time, hunting, nevertheless, continues to be integral with almost all other aspects of human and social life. This does not mean that all able-bodied men are hunters, or that all families are hunting families. At the beginning of the *Piniariarneq* project in 2015, there were about 60 occupational hunters in Avanersuaq. While life as a hunter does continue to catch the interest and passion of some young men in Avanersuaq and elsewhere in Greenland, many, like their female peers are attracted by the prospect of education in the South (Flora 2015). This trend is exacerbated by the fact that aspiring hunters need the support of a wife who is willing to live and work as a hunter’s wife, rather than embarking upon education or a professional career.

Whereas subsistence hunting and cash economy are kept as two mutually exclusive realms by lawmakers in parts of the Canadian Arctic, this is not the case in Greenland, neither conceptually nor in practice. In fact, one might argue that subsistence hunting in Greenland could not prevail (subsist) were it not for its intertwinement with cash economy and the international market (Dahl 2000; Wenzel 2000; Hastrup 2015). In contrast to part-time or recreational hunters, occupational hunters must maintain hunting licenses that permit them to hunt quota-regulated animals, such as narwhal, beluga (*Delphinapterus leucas*), polar bear, and walrus (*Odobenus rosmarus*) (see also Andersen et al. 2017). In practical terms, this implies that at least 50% of their taxable income should come from hunting activities. Occupational hunters are thus obligated to trade in at least part of their catch. According to hunters who participated in the *Piniariarneq* project, their main sources of cash income are narwhal *mattak*, Greenland halibut (*Reinhardtius hippoglossoides*), as well as by-products such as narwhal and walrus tusks, sealskin, and for some polar bear claws and skins, the latter also being essential for men’s trousers. Avanersuaq is among the areas in Greenland with the lowest gross household income (Statistics Greenland 2017,6), and according to officials at the local council office, as well as the head of the local hunters’ association (KNAPP), some hunting families live below the poverty line for parts of the year. Much of a household’s financial income needed to meet expenses associated with maintaining hunting equipment—purchasing fuel and various household costs—comes from the hunter’s wife, supplemented sometimes by relatives. Many households in Avanersuaq today that are not directly engaged in hunting activities rely on shares or being able to buy hunted food from the occupational hunters. Hunting, employment, and cash are part of the same livelihood; and subsistence, therefore, does not relate merely to the hunting activity itself, but to the whole community.

## The *Piniariarneq* study

The main purpose of the *Piniariarneq* study was to map the distribution and seasonality of resource spaces through the movements of occupational hunters in present day Avanersuaq. For a detailed description of the project, and the cross-disciplinary, collaborative effort on which it is based, we refer to Andersen et al. (2017).

**Fig. 1:**
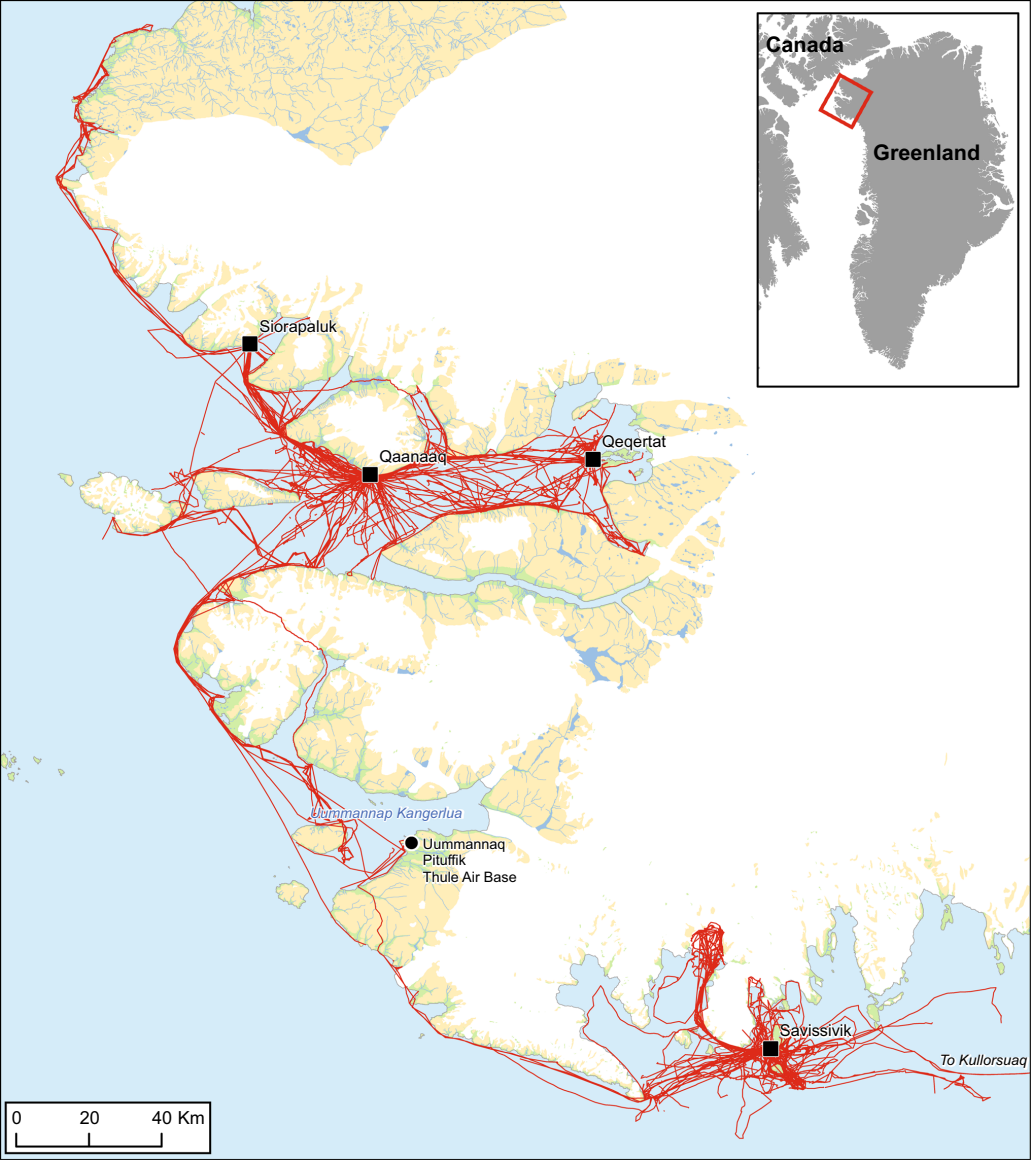
All GPS tracks from hunting trips recorded through the *Piniariarneq* study (*n* = 725) from May 16th 2015 to June 26th 2016. The tracks are a result of both dog sledge and motor boat traffic, as well as trips on foot. Notice how the towns in the northern part of the area are tightly interconnected, whereas Savissivik is not directly connected to any of the other towns. Uummannap Kangerlua (Wolstenholme Fjord), which was the heart of the district during the Thule Station period, almost takes on the appearance of a void in the motor boat/dog sledge traffic network of today

The project began in earnest in May 2015, when 19 occupational hunters from Avanersuaq agreed to track their hunting trips during a full year using a custom-made application (app) installed on a handheld GPS device. Named *Piniariarneq* (hunting trip), the app was designed to capture detailed information on individual hunting trips, which beyond the route itself, included means of transportation, the composition of the hunting party, catches, and observations of animals, as well as anything else the hunter would find interesting and relevant to document through geotagged written notes, photographs, and video footage (Andersen et al. 2017). In total, we distributed 19 GPS units and received data from 15 hunters from Qaanaaq and two from Savissivik (Fig. 1). Though two of the participating hunters from Qaanaaq had strong ties to Siorapaluk, and regularly went on hunting trips with fellow hunters from there, no hunters actually resident in Siorapaluk were involved. The tracking lasted a little over a year, from 16th of May 2015 to 26th of June 2016. During this period, approx. 167 000 GPS positions from hunting trips were recorded, covering a distance of 700 km from 73.5N (northern Upernavik) to 78.5N (Inglefield Land). Underway, 855 catches and observations of animals were registered, distributed across 33 different species. Furthermore, almost 2900 geotagged photos and videos document the in situ activities of the hunters in the landscape (see Andersen et al. 2017 for examples). It is on basis of this unique record of Inughuit hunting practices, collected by the occupational hunters themselves, that we base our description of resource spaces and seasonal rhythms in contemporary Avanersuaq.

### Networks and mobility

The GPS positions from the hunting trips translate into approx. 20 000 km of tracks, which weave Avanersuaq into an intricate network structure connecting permanent settlements, hunting camps, and hunting grounds in the landscape (Fig. 1). Qaanaaq and Savissivik, the homes of the tracked hunters, constitute clear hubs in the network. However, it is also evident that the northern villages, Qaanaaq, Qeqertat, and Siorapaluk are tightly interconnected, whereas connections from Savissivik to other permanently inhabited towns and villages are oriented southwards, out of Avanersuaq to Kullorsuaq, Nuussuaq, and Nutaarmiut. There are no direct connections between the northern villages and Savissivik, and only once, at the old Uummannaq settlement in Uummannap Kangerlua (Wolstenholme Fjord), do the tracks from north and south intersect during the celebration of Armed Forces Day at the adjacent Thule Air Base in Easter 2016. Thus, based solely on these GPS tracks, one cannot escape the impression of an area broken into two with Uummannap Kangerlua constituting a void in what Rasmussen once referred to as the centre of the district (Rasmussen 1921).

Almost all routes are confined to the sea and only in three instances do we observe more extensive crossings over land. Thus, all movements are in one way or another premised by the relation between sea ice and open water, in particular the extent of land-fast ice. In 2015, when the *Piniariarneq* project commenced, the open water season began with the breakup of the land-fast ice in the early July (Fig. S1). Sea-ice formation began in October, but it was not until the middle of November that land-fast ice suitable dog sledge traffic started to form. We see this directly reflected in the means of transportation recorded by the hunters (Fig. S2). From November to June, the dog sledge appears to have been the dominant means of transportation, whereas the hunters almost exclusively used motor boats between July and October. In May and June, the hunters use a combination of dog sledge and motor boat: the motor boat is transported on dog sledge to the edge of the land-fast sea ice to be used there during the ice edge hunt. Thus, the motor boat is important for at least 6 months of the year. Hunting trips by foot are recorded throughout the year, however, with a tendency to peak in October–November during the transition period between the motor boat and dog sledge seasons.

As for the overall mobility in the landscape, there is a tendency towards a bi-modal distribution across the months of the year with regard to both total trip length and duration (Fig. S3). There is a marked peak of mobility in July, corresponding to the beginning of the motor boat season, and we see another peak in February–March associated with dog sledge traffic on the land-fast ice. Mobility is a little lower during spring, where traffic is more or less restricted to transport to and from ice edges located close to the towns. The most striking feature of Fig. S3 however, is the plummeting of mobility in October–December. This corresponds to the period of sea ice formation and the gradual arrival of the polar night. At this time, there is too much new ice to navigate the waters by motor boat, yet stable fast ice suitable for dog sledge traffic has not yet formed. Thus, while the bi-modal mobility pattern is a testament to the success of Inughuit hunters in coping with two radically different landscapes by shifting mode of movement, it also serves to underline the challenges associated with the transition periods, especially the slow sea-ice formation during autumn. In November 2015, when mobility was at its lowest, more than 40% of all hunting trips were undertaken by foot (Fig. S2).

### The dynamics of resource spaces through a seasonal cycle

**Fig. 2:**
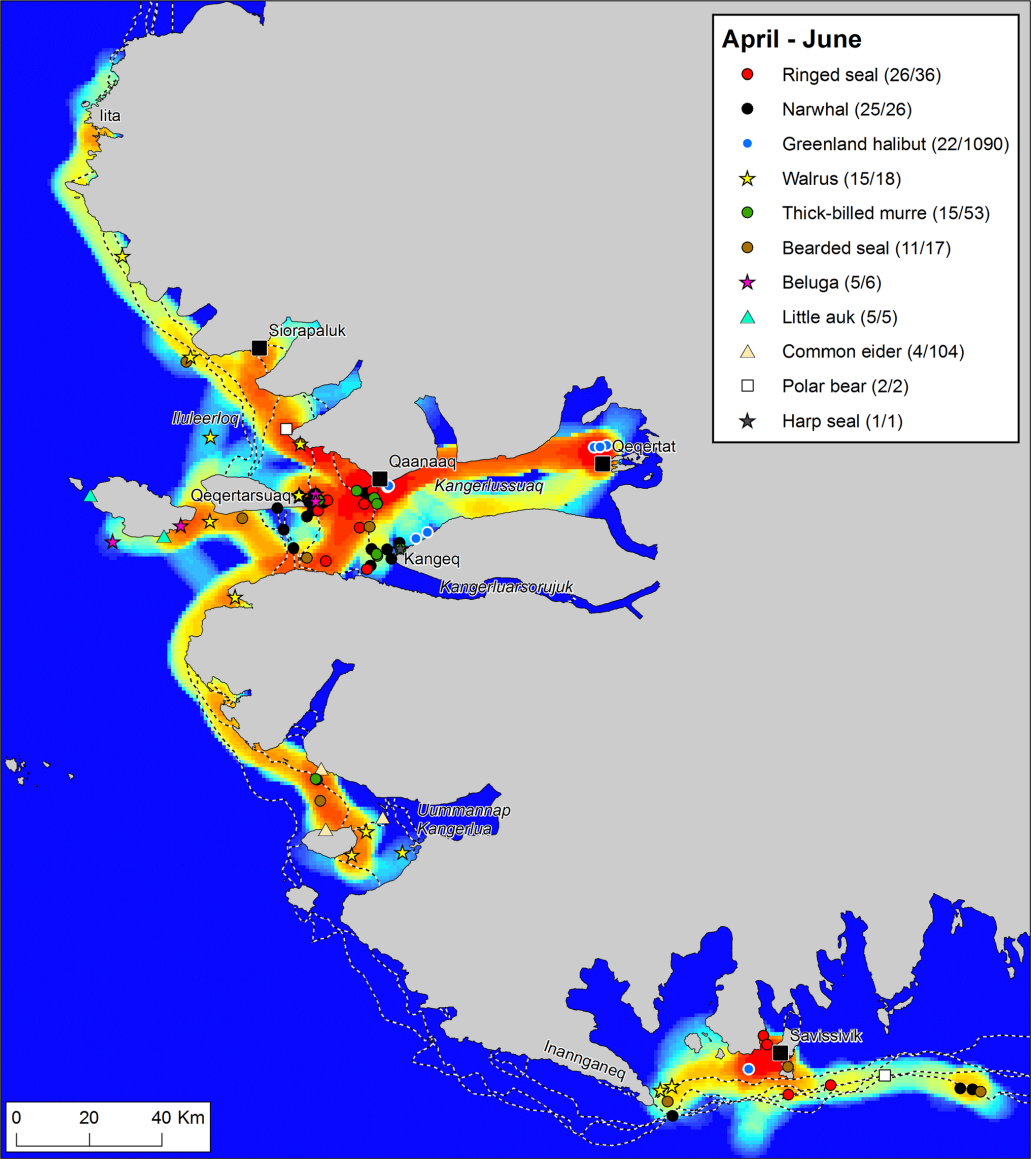
Traffic intensity and catches during the months April–June 2015/16, recorded by hunters that participated in the *Piniariarneq* study. Traffic intensity (km track line per km^2^) is displayed on a relative scale for the season, ranging from blue (low) over yellow (intermediate) to red (high). In the legend, the number of recorded catch events and the estimated number of individuals bagged are given for each species. Place names mentioned in the main text are indicated on the map. The dotted white-and-black lines represent the extent of the land-fast ice during the first week of the months April–June 2015/16. This was clearly a period dominated by the hunting of a wide range of species along the edge of the land-fast sea ice

**Fig. 3:**
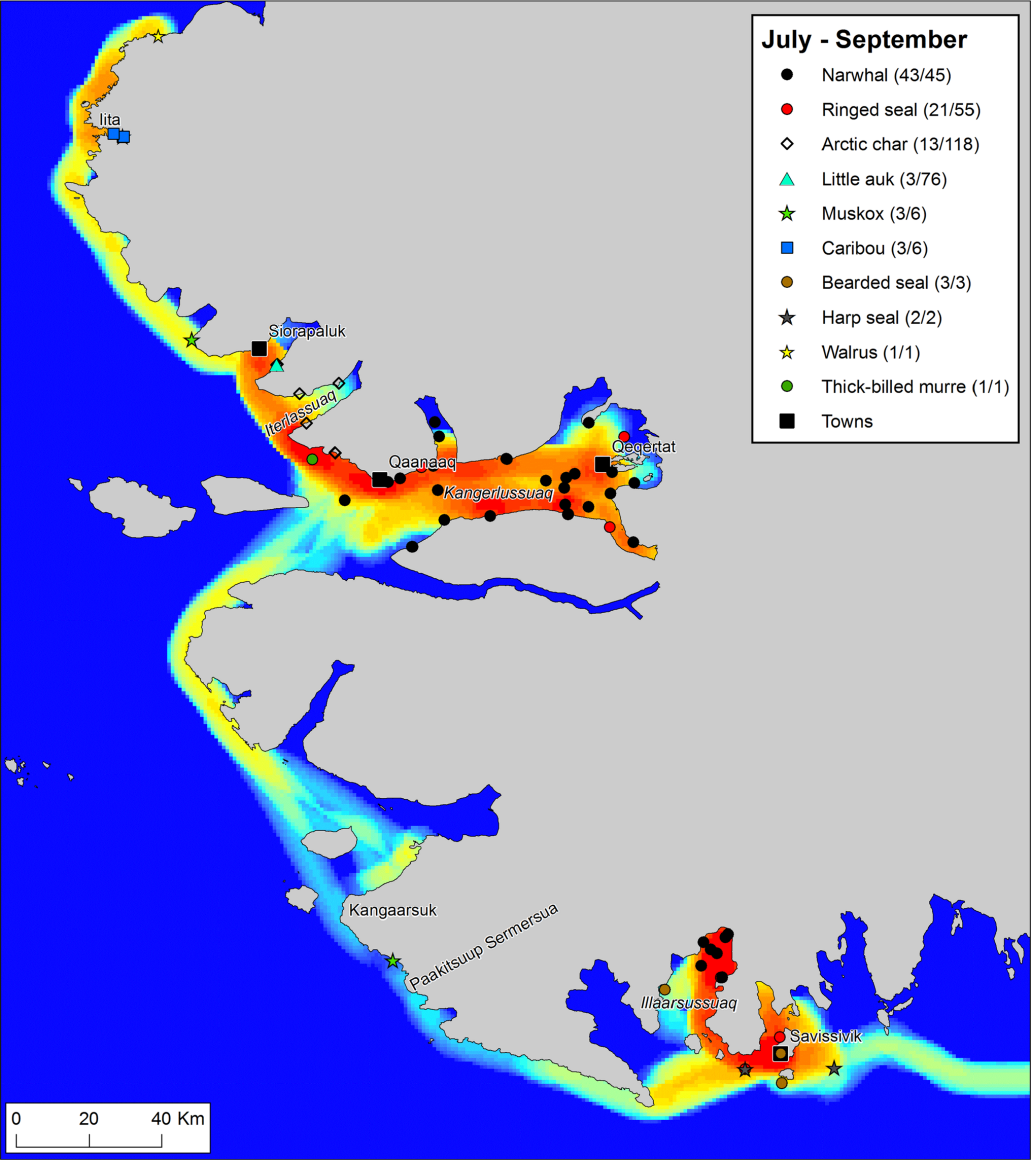
Traffic intensity and catches during the months July–September 2015 (“the open water season”), recorded by hunters that participated in the *Piniariarneq* study. Traffic intensity (km track line per km^2^) is displayed on a relative scale for the season, ranging from blue (low) over yellow (intermediate) to red (high). In the legend, the number of recorded catch events and the estimated number of individuals bagged are given for each species. Place names mentioned in the main text are indicated on the map. This period was dominated by open water hunting of narwhals in Kangerlussuaq (Inglefield Bredning) and Illaarsussuaq (Sidebriksfjord)

To explore the dynamics of resource spaces through a seasonal cycle, we have broken down the year of *Piniariarneq* data in four seasons, based primarily on the sea-ice dynamics. For each season, we have mapped the traffic intensity of the hunters by calculating km route per km^2^ within a radius of 5 km of every point in the landscape, and combined this with plots of recorded catches (Figs. 2, 3, 5, 6). This gives a picture of important traffic corridors, intensively used areas, and the actual activities taking place in different parts of the landscape throughout a seasonal cycle.

#### Ice edge season: April–June

At this time of year, when the light has returned and the land-fast ice is still extensive, the movement of the hunters is clearly directed towards the ice edge (Fig. 2). This is the period of ice edge hunting. At Savissivik, movements and catches are mainly concentrated in an approx. 100 km long, east–west oriented band, ca. 15 km south of the town, corresponding closely to the position of the ice edge in both 2015 and 2016. Local hotspots along the ice edge are indicated at the tip of Inannganeq (Cape York) and at the far eastern end of the range in conjunction with a pronounced underwater ridge. With respect to Qaanaaq hunters, movement is directed towards southwest with several multi-species hotspots within a range of 25 km from the town. The multiple clusters of catches here are owed to differences in the position of the ice edge between 2015 and 2016; the ice edge was located closer to the town in 2016 (Fig. S1). Thus, in the spring of 2015, the ice edge hunt was mainly concentrated off the eastern tip of Qeqertarsuaq (Herbert Island), whereas in 2016, concentrations of catches are evident both off Kangeq at the mouth of Kangerluarsorujuk (Olrik Fjord) and in an area only 5 km south of Qaanaaq. Besides the local ice edge hunting, there is also evidence of long-range hunting trips at this time of the year. Hunters from Qaanaaq have undertaken trips south to Uummannap Kangerlua (Wolstenholme Fjord) and north to Anoritooq in pursuit of walrus. In April and May, we also observe a continuation of the important winter fishery for Greenland halibut centred in Kangerlussuaq (Inglefield Bredning) near Qeqertat and Qaanaaq.

With respect to the catches, the most striking feature is the considerable diversity of species (Fig. 2). Clearly, many and important game animals are taken at this time of the year. Most prominent is, perhaps, the narwhal. However, the walrus, which has not yet left the area for the summer and are taken in the drift ice over the shallow areas (typically < 100 m) in Uummannap Kangerlua (Wolstenholme Fjord) and Iluleerloq (Murchison Sound), also make up a significant portion of the hunting bag and directly influence the movement pattern of the hunters by inspiring to long trips. Beluga and polar bear are also caught, and besides the omnipresent ringed seal (*Phoca hispida*), we also note hunting of seabirds that have arrived for the summer breeding season.

#### Open water season: July–September

**Fig. 4:**
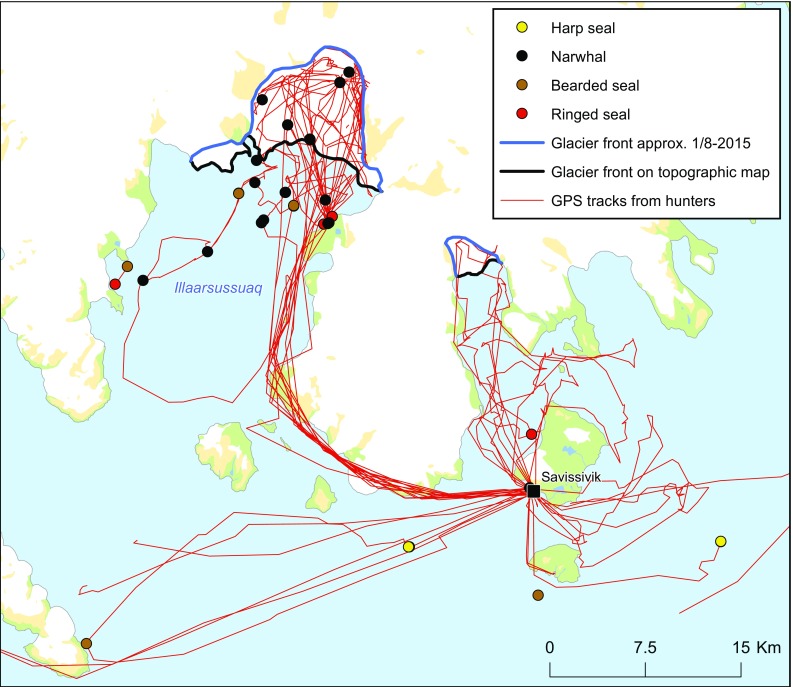
GPS tracks and animal recordings (both catches and sightings) from July to September 2015, documented by two hunters from Savissivik who participated in the *Piniariarneq* study. The difference between the glacier fronts as of August 1st 2015 (in blue), and the glacier fronts charted on the topographic map (in black), demonstrates that the glacier in the bottom of Illaarsussuaq (Sidebriksfjord) has retracted more than 7 km, opening up the area in which the narwhals were hunted during the open water season of 2015. The narwhals did not return Illaarsussuaq in the summer of 2016

The onset of the open water season is clearly associated with a complete shift in the orientation of the hunters in the landscape (Fig. 3). Whereas in the spring, the hunters traveled “outwards” towards the ice edge, the hunters now travel “inwards”, into the bottom of the fjords. Thus, spring and summer resource spaces are almost complementary. In terms of the hunting bag, there is still a large variety of species on the list. However, the recordings are concentrated on one single species, the narwhal, which, without doubt, is the main driver behind the shift in the hunters’ use of the landscape. Hunters from Qaanaaq pursue the narwhals in the inner parts of Kangerlussuaq (Inglefield Bredning). Hunters from Savissivik usually catch summering narwhals in Qimusseriarsuaq (Melville Bay) (Heide-Jørgensen et al. 2013), but as shown by the GPS tracking, 2015 was different. In 2015, the narwhal hunt from Savissivik was concentrated in Illaarsussuaq (Sidebriksfjord) only 30 km from the village. One explanation for this could be an earlier or more complete breakup of the land-fast ice than usual, allowing the narwhals to penetrate into the fjord. However, a closer look at Illaarsussuaq reveals that significant changes have taken place in this area also on a longer timescale (Fig. 4). Since the glacier of the topographic map was charted, the glacier front has retracted more than 7 km, opening up the area in which the narwhals were encountered in 2015. According to one hunter, the retreat of the glacier has taken place over the last 3 decades. The narwhals did not return to Illaarsussuaq in 2016, and this may thus illustrate how a combination of long-term trends (the melting of the glacier due to the warming of the Arctic) and year-to-year variation (in ice cover and/or possibly food availability to the narwhals) introduces dynamics in the distribution of resource spaces. Such dynamics may have important consequences for the hunters, in this case not least because of the significantly lower fuel costs of traveling from Savissivik to Illaarsussuaq, as opposed to Qimusseriarsuaq.

Aside from the narwhal hunting, we also note other activities in the landscape during the open water season. Little auks (*Alle alle*) are caught in the large breeding colonies in vicinity of Savissivik and Siorapaluk, and Arctic char (*Salvelinus alpinus*) are procured at the outlets of several rivers, mainly in Iterlassuaq (MacCormick Fjord). In September, hunters from Qaanaaq have made a boat trip all the way to Etah to hunt caribou and muskox, whereas Savissivik hunters pursue muskox in the lush little auk colonies between Kangaarsuk (Cape Atholl) and Paakitsuup Sermersua (Pituffik Glacier) (see Mosbech et al. 2018).

#### Season of sea-ice formation: October–December

**Fig. 5:**
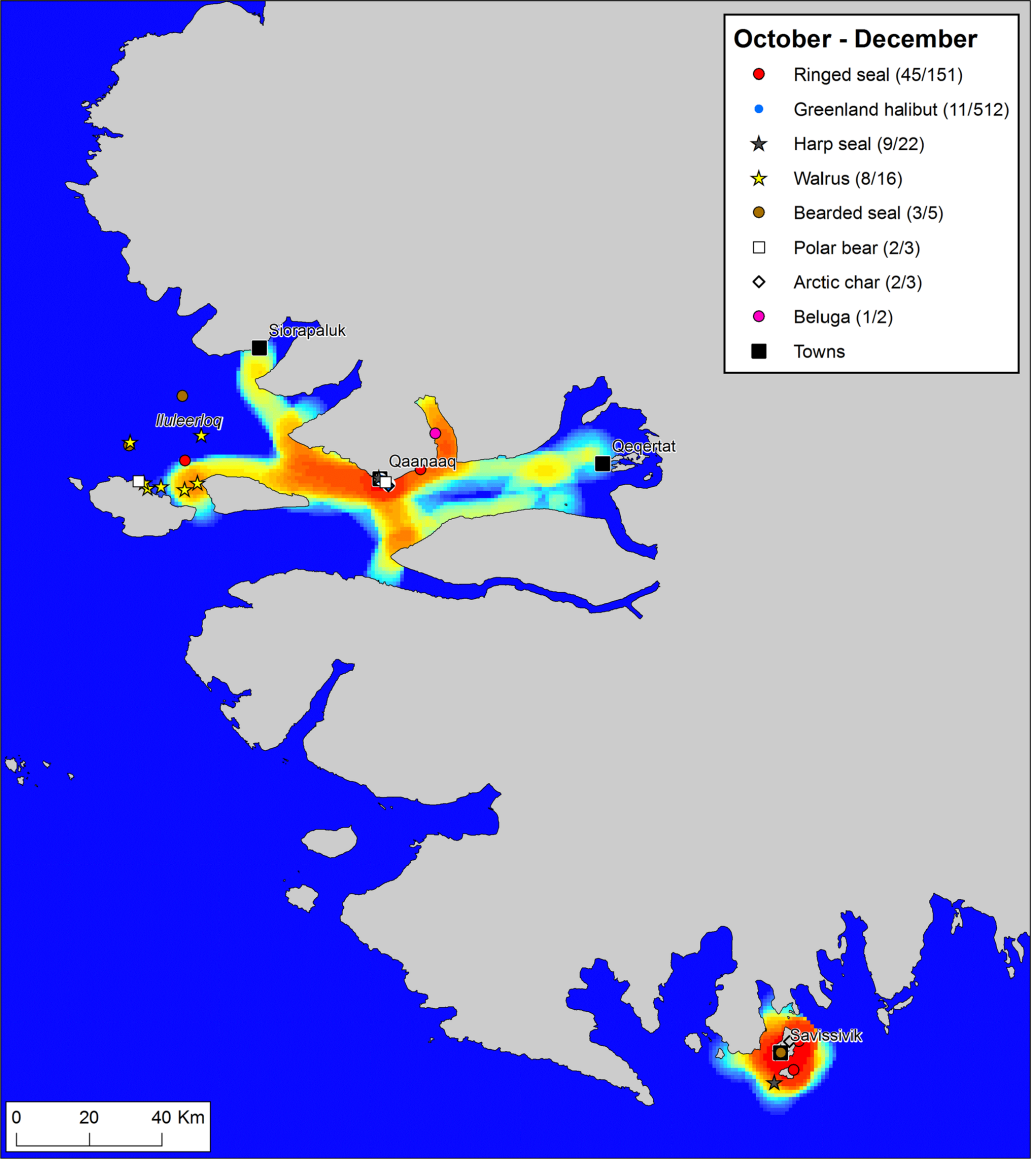
Traffic intensity and catches during the months October–December 2015, recorded by hunters that participated in the *Piniariarneq* study. Traffic intensity (km track line per km^2^) is displayed on a relative scale for the season, ranging from blue (low) over yellow (intermediate) to red (high). In the legend, the number of recorded catch events and the estimated number of individuals bagged are given for each species. Place names mentioned in the main text are indicated on the map. During this period of gradual sea-ice formation, mobility was restricted and most hunting activities took place close to the towns. However, hunters from Qaanaaq made a few longer trips to Iluleerloq (Murchison Sound) to hunt walrus

During autumn, when the sea ice starts to form and the days darken, the resource spaces of the hunters seem to contract (Fig. 5). The new ice gradually puts a stop to motor boat traffic, yet stable fast ice suitable for the dog sledge does not form until the end of the period (Fig. S1). Consequently, the movement of the hunters is now centred on the permanent settlements and limited in extent. Especially in Savissivik, all movement is confined within a radius of 15 km from town.

The hunting bag also seems to shrink, both in terms of the diversity of species and the sheer size of the catch in kg. In Savissivik, activities are focused on netting of seals just outside town. In October, both harp seals (*Pagophilus groenlandicus*) and ringed seals are taken in the nets, in November–December only ringed seals. In Qaanaaq, Greenland halibut fishery just 3 km from town seems to become important towards the end of the period. However, in October, the Qaanaaq hunters do embark on longer trips in pursuit of the walrus, which has now returned from Canada to winter in the shallow waters of Iluleerloq (Murchison Sound). This hunt is based on motor boats, and the hunters thus make use of the narrow window of opportunity presented to them by the overlap between the tail end of the motor boat season and the arrival of the walrus. This hunt in Iluleerloq is important, not least because it secures dog food and thereby helps making winter mobility possible.

#### Fast ice season: January–March

**Fig. 6:**
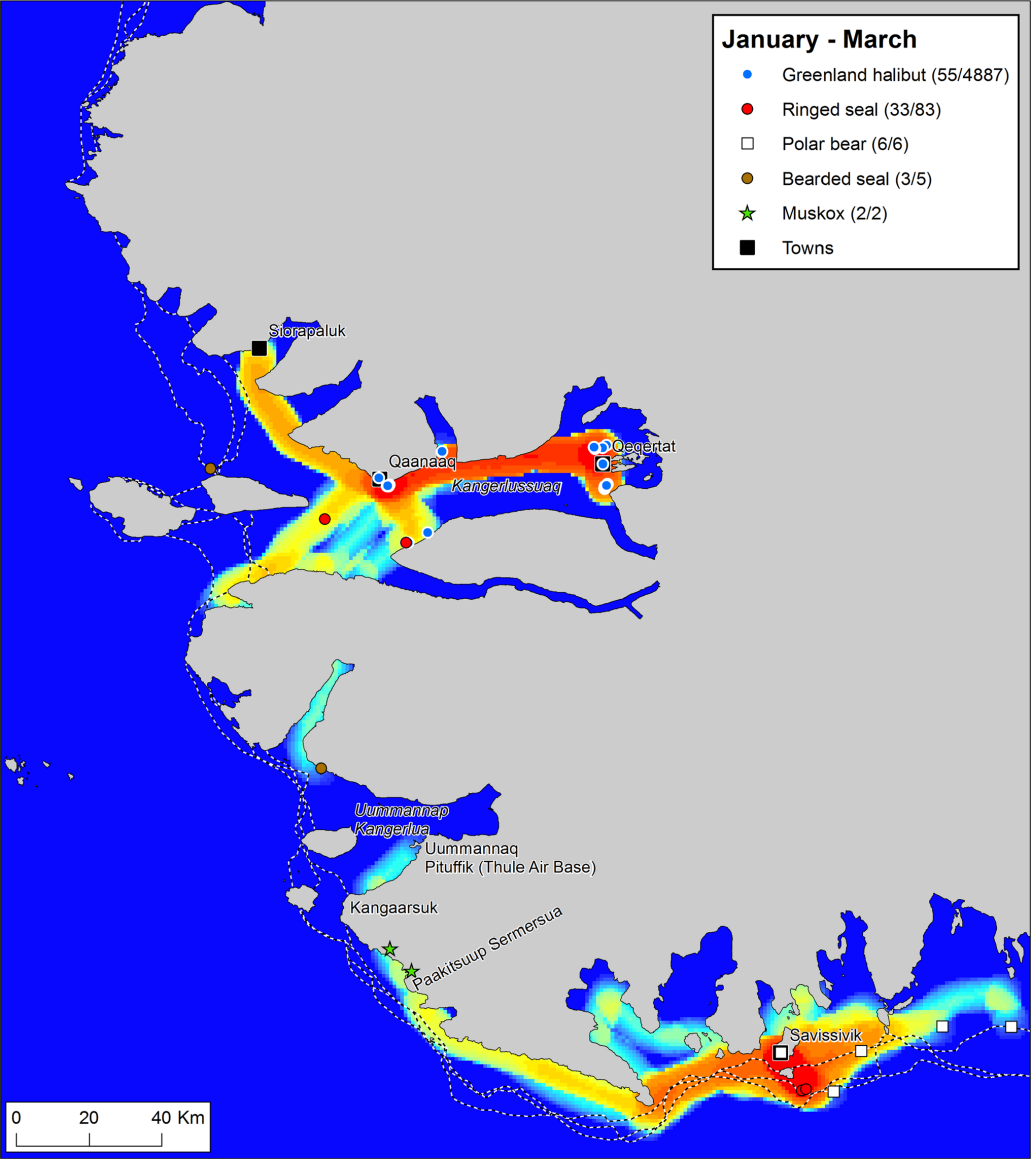
Traffic intensity and catches during the months January–March 2016, recorded by hunters that participated in the *Piniariarneq* study. Traffic intensity (km track line per km^2^) is displayed on a relative scale for the season, ranging from blue (low) over yellow (intermediate) to red (high). In the legend, the number of recorded catch events and the estimated number of individuals bagged are given for each species. Place names mentioned in the main text are indicated on the map. The dotted white-and-black lines represent the extent of the land-fast ice during the first week of the months January–March 2016. During this period of extensive fast ice, activities of Qaanaaq hunters were focussed on Greenland halibut fishery in Kangerlussuaq (Inglefield Bredning), whereas Savissivik hunters netted seals close to town and went on long trips towards east in pursuit of polar bears

With the fast ice now formed and suitable for dog sledge traffic, we see a massive expansion in the movement of the hunters (Fig. 6). Towns are re-connected and hunters from both Qaanaaq and Savissivik travel all the way to the old Uummannaq settlement in Uummannap Kangerlua (Wolstenholme Fjord) to attend Armed Forces Day at Thule Air Base. Resource spaces generally expand, but in very different ways for Qaanaaq and Savissivik hunters.

In Kangerlussuaq (Inglefield Bredning), activities appear to be completely focused on long-line fishery for Greenland halibut. Permanent fishing camps are established on the ice, primarily outside Qeqertat and Qaanaaq, but also at several other places in the fjord. At these sites, the hunters have recorded catches of Greenland halibut throughout the period from December to May, with a peak in February. Especially, at the fishing sites north of Qeqertat, large quantities of Greenland halibut are landed, and the intensive traffic between Qaanaaq and Qeqertat is a result of transport of this catch, which is traded in Qaanaaq.

In Savissivik, not a single Greenland halibut catch was recorded. Instead, the netting of ringed seals close to town seems to have continued throughout the winter and constituted a primary activity. However, we also see long trips towards southeast, where an important polar bear hunt takes place on the fast ice close to the ice edge. Thus, already during winter, the ice edge is becoming somewhat important for Savissivik hunters. The muskox that over-winter in the little auk colonies between Kangaarsuk (Cape Atholl) and Paakitsuup Sermersua (Pituffik Glacier) were also hunted.

### Regional differences and settlement pattern

Having explored the resource spaces of the hunters through a seasonal cycle, some overall patterns emerge. First, hunting practices in contemporary Avanersuaq are by no means homogeneous. In Qaanaaq, Greenland halibut is the most recorded species amongst the catches, whereas ringed seal ranks third (Fig. S4). In Savissivik, ringed seal completely dominates with more than 60% of the recordings, whereas Greenland halibut is virtually absent. This difference is mainly due to divergent autumn and winter strategies, Greenland halibut fishery vs. seal netting, whereas spring and summer hunts are much alike in the two places. More subtle differences are also apparent, e.g., relatively more walrus recordings in Qaanaaq and a more prominent place of polar bears and harp seals in Savissivik. It is important to bear in mind that this comparison represents the sum of recordings in the two towns, and that considerable variability also exists between individual hunters from the same town.

**Fig. 7:**
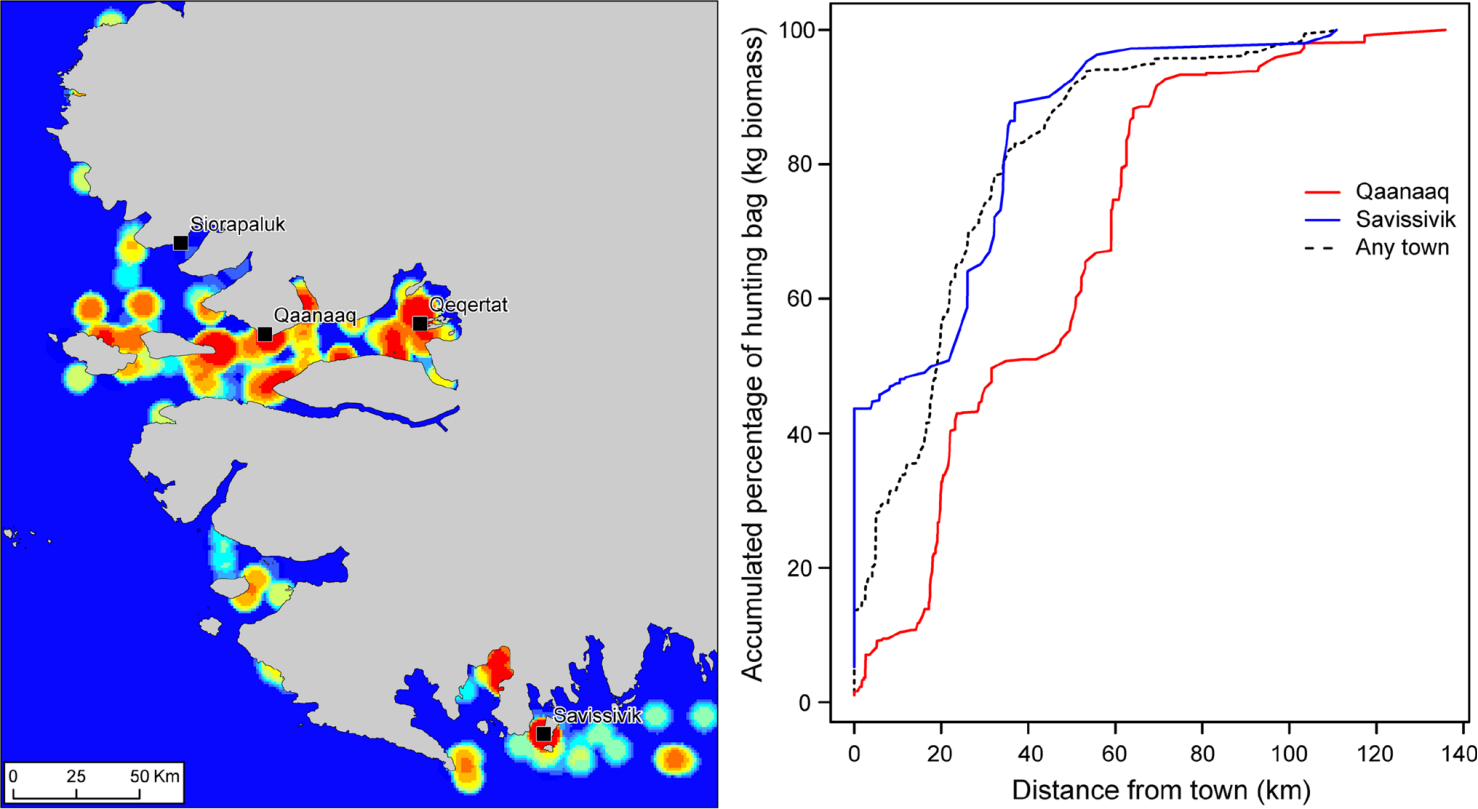
Spatial distribution of the total biomass of catches (in kg) recorded by the 17 hunters who participated in the *Piniariarneq* study between May 16th 2015 and June 26th 2016. *Left panel:* On a colour scale from blue (low) to red (high), this map shows kg biomass harvested per km^2^ within a radius of 5 km around every point in the landscape. It thus highlights hotspots of resource extraction seen over the whole GPS tracking period. South and southwest of Qaanaaq several hotspots are apparent, mainly resulting from the spring ice edge hunt but also from Greenland Halibut fishery during winter. The large hotspot close to Qeqertat is a combined result of the summer narwhal hunt and the winter fishing for Greenland halibut. The walrus hunt in Iluleerloq (Murchison Sound) is also clearly visible. The hunting from Savissivik mainly resulted in two hotspots, one in Illaarsussuaq (Sidebriksfjord) corresponding to the summer narwhal hunt, and one centred on the town relating to seal netting during winter. However, several smaller hotspots are also apparent along the ice edge. *Right panel*: This graph shows the accumulated percentage of the hunting bag (in kg) as a function of distance from the hometowns of the participating hunters (red line: Qaanaaq; blue line: Savissivik), and any town in the study area (dashed line). As can be seen, Qaanaaq and Savissivik hunters bagged 75% of their gross weight of game within approx. 60 and 35 km of their hometowns, respectively

Second, it is evident that the settlement pattern has a strong structuring effect on the resource spaces of the hunters. From a strictly biological perspective, there are living resources to be harvested at many places in the landscape, yet only in relative proximity to permanent settlements are the resources harvested intensively (Fig. 7). For Qaanaaq hunters, approx. 75% of the biomass of the hunting bag was procured within distance of 60 km from their hometown. The two hunters from Savissivik bagged 75% of their gross weight of game within a mere 35 km from town. In Savissivik, 2015 was an unusual year due to the narwhal hunt taking place close by in Illaarsussuaq (Sidebriksfjord), not in Qimusseriarsuaq (Melville Bay), but this does not change the general picture. The hunters of Avanersuaq hunt in relative proximity to home, and with the population concentrated in only four permanent villages today, their procurement of living resources in the landscape is, therefore, constrained to a relatively small area. This does not mean that long hunting trips are never undertaken, or that resources procured at distant hunting grounds are unimportant. Indeed, life in Qaanaaq would be poorer without caribou from Etah. However, rather than an extensive use of a large part of the landscape, it seems that a hallmark of contemporary hunting in Avanersuaq is intensive use of a rather small portion of the landscape in proximity to permanent settlement.

## Historical perspectives on resource spaces

A complex interplay of processes—some continuous, some abrupt and drastic—have set in motion a variety of changes in Inughuit hunting practices through time. Juxtaposing the findings of the *Piniariarneq* project with historical records from Avanersuaq allows us to contextualize the current use and formation of resource spaces in a longer time perspective. For analytical purposes, we will focus on two periods, referred to here as the “Thule Station Period” and the “Post-Thule Station Period”. The Thule Station Period (Rasmussen 1921; Vibe 1950; Holtved 1967; Grønnow 2016) begins with the establishment of Knud Rasmussen’s trading station in North Star Bay in 1910. It ends in 1953, when, as a consequence of the establishment of the American Thule Air Base, the station was closed and the inhabitants of the large adjacent settlement Uummannaq were relocated to Qaanaaq. During the Thule Station Period, the economy of the Inughuit was driven by the trade in fox fur—a development that was reinforced in the early 1930s by the establishment of two additional trading posts in the northern- and southernmost parts of the district. While the Thule Station had a clear beginning and end (1910–1953), and, in some respects, can be regarded as marker of a historical “period”, this is much less the case for the time that followed. By “Post-Thule Station Period” (ethnographic information compiled in Gilberg 1971 and 1984), we refer to the time span from 1953 up until the mid-80s, when the prices of sealskin began to plummet. It thus covers the time of the new settlement patterns and resource spaces that characterized the decades following the establishment of Qaanaaq as the centre of the region and a number of permanent villages.

### Changes in networks, mobility, and settlement patterns

**Fig. 8:**
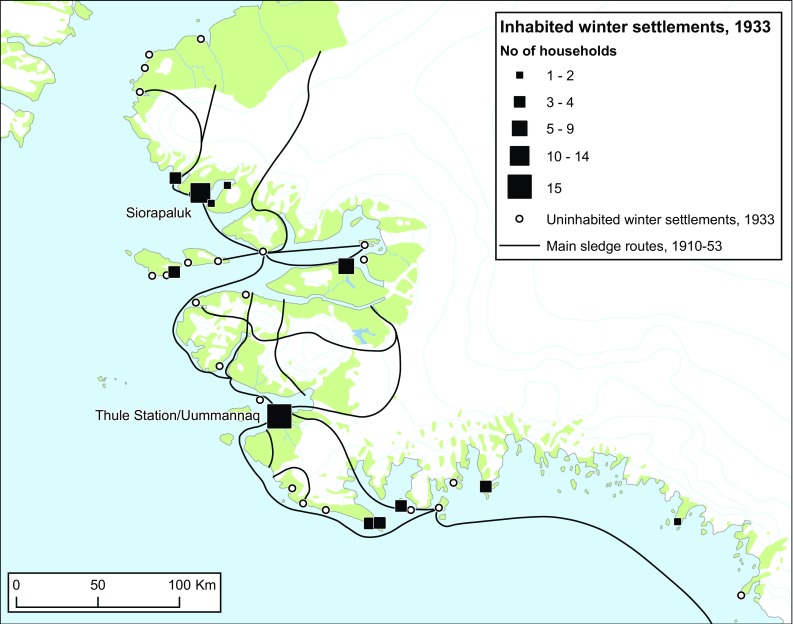
Active winter settlements and their number of households in 1933. Marked concentrations of families are seen at the Thule Station itself and at the newly established trading post Siorapaluk. The black lines represent the main sledge routes during the Thule Station Period, 1910–1953. Note that several routes lead across fringe areas of the Inland Ice. They served partly as shortcuts, partly as ‘escape routes’ in case of open water, unstable sea-ice conditions or lack of an ice foot at the coast. Reproduced with Permission from Holtved (1935, 1937); Holtved (1944); Gilberg (1971); Grønnow (2016)

During the Thule Station Period, we encounter networks of transportation and settlement patterns that are fundamentally different from the present. Typically, the hunting families changed winter residence every second or third year within a network of thirty-six preferred winter sites. Of these sites, 10–15 were in use at the same time, each occupied by two–four families (Fig. 8). The settlement of Uummannaq, immediately north of the Thule Station, grew from 3 to 15 households, but we should note that people moved through (rather than to) the village, typically staying only for a couple of winters at a time (Holtved 1944: 13–14) (Fig. 8). The two trading posts established in the early 1930s, Savissivik and Siorapaluk, also attracted people. Early spring aggregation sites, where many families gathered for communal walrus hunting, were situated at Neqe and Pitoqarfik north-west of Siorapaluk.

The Thule Station Period became the hey-day of dog sledge transportation as a result of ample supplies of trade goods (including wood) from the station and, owing to the fox fur trade, a remarkable increase in Inughuit purchasing power (Grønnow 2016: 3). Drawn by teams of 15 dogs, the sledges were bigger than ever before and they carried heavy loads of entire families with all their gear and food supplies, as well as quantities of meat and blubber between caches and settlements (Holtved 1967: 62). A dense web of sledge routes covered the entire district (Fig. 8).

Compared to today, Inughuit residential mobility during the Thule Station Period was extremely high. Households moved winter residence regularly, and the hunters thus accumulated first-hand knowledge of resource spaces in the entire region. The high mobility facilitated both maintenance and renewal of social relations (Aporta 2009; Grønnow 2016: 2–4; Mauss 1979). Sometimes, the web of sledge routes expanded as hunting groups or families crossed the ice of Smith Sound, undertaking long distance hunting expeditions to Umimmatooq (Ellesmere Island) (Rasmussen 1921: 561–562).

Many sledge routes during this period went across inland areas and over glaciers (Fig. 8). These routes, some of which are inaccessible today due to unstable snow cover and retreat of glaciers, were important shortcuts between settlement areas and, not least, escape routes that prevented isolation and famine if the sea ice suddenly broke up or the ice foot washed away. The few motor boats in the area were owned by the Thule Station, and used mainly to assist hunters from Uummannaq (Holtved 1937: 80).

Both settlement pattern and mobility changed in the Post-Thule Station Period. The forced relocation of the Uummannaq population to Qaanaaq in 1953 gave this place status as town and new administrative centre of the region. By 1969, Qaanaaq had 35 households (245 inhabitants or 40% of the total population of 603 individuals in the district). The two minor trading posts grew into villages with 14 and 17 households, respectively. Interestingly, a number of old settlements were “revived” in the wake of the 1953 event: wooden standard houses were built in Qeqertarsuaq (1953), Qeqertat (1953), Moriusaq (1963), and Narsaarsuk (c. 1965) (Gilberg 1971: 176).

**Fig. 9:**
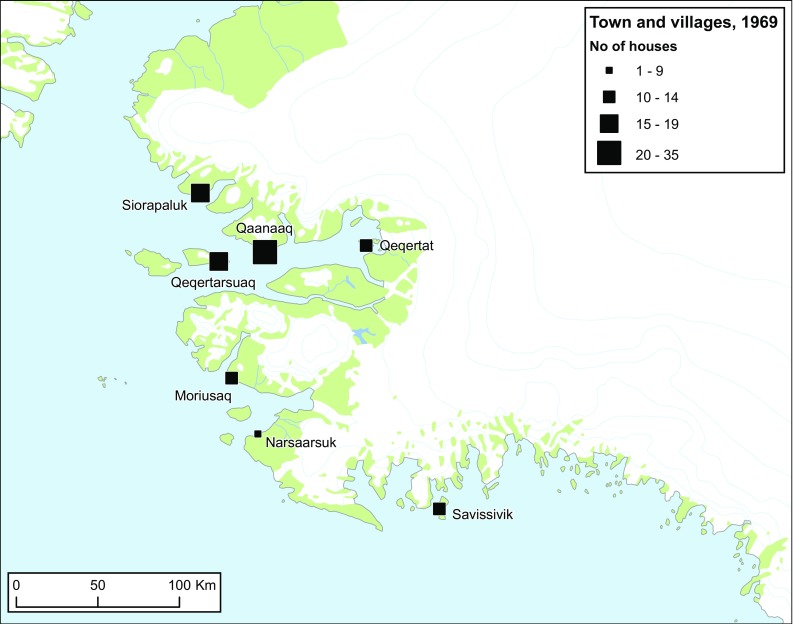
Permanently inhabited villages and their number of houses in 1969. The distribution of houses during the Post-Thule Station Period shows a marked concentration of Inughuit families in Qaanaaq and the nearby hamlets Siorapaluk and Qeqertarsuaq. Reproduced with Permission from Gilberg (1971); Grønnow (2016)

This new settlement pattern, consisting of a main town and six permanently inhabited villages (Fig. 9), shaped a web of transportation routes that by the early 1960s still resembled the network of the Thule Station Period. In particular, Moriusaq with its ten households (in 1969) and shop came to be an important hub, owing to the rich hunting grounds in the fjord and access to material resources from Thule Air Base. The main traveling routes on the sea ice, and not the least the “shortcuts” crossing the ice cap were maintained. Several hunters’ cabins were erected supporting the sledge route network that was based on the stable fast ice conditions and long sledge seasons, which still prevailed during this period. During the Post-Thule Station Period several hunters came to own boats, typically small wooden cutters (Danish: *nummerbåde*). These served as support for kayaks on narwhal and walrus hunts and connected the villages during the brief open water period (e.g., Gilberg 1971: 16; Ivik 1992: 46).

We have seen how changes in routes are shaped by changing sea ice and snow conditions, introduction of new transportation technologies as well as changes in settlement patterns. In turn, the primary drivers of important changes in settlement patterns during the two historical periods were external political/economic factors, such as the establishment of trade stations and permanent villages. Obviously, the establishment of Thule Air Base marked a critical transition. The former pattern with frequent movements between winter residences situated in all parts of the district changed radically and abruptly into a settlement pattern with a focal town, Qaanaaq, and six villages equipped with permanent wooden standard houses. As the Post-Thule Period progressed, this changed into the picture that we see today with Qaanaaq as the all-dominant centre of the district (79% of the total population of the region in 2016) and only three other villages. Of these, the southernmost village, Savissivik, separated from the other villages by great distances, has experienced increasing isolation and de-population (number of inhabitants, 1977: 129; in 2016: 56) (Statistics Denmark 1953–1984).

Despite their differences, the transportation network of both historical periods described above reflected a ‘landscape of dog sledges’—in contrast to the present, where the travel routes as mapped by the hunters during the *Pinariarneq* project to a large degree reflect a ‘seascape of motorboats’.

### Long-term changes in seasonal resource spaces

#### Spring resource spaces

The time span from the Thule Station Period to the present is characterized by remarkable shifts concerning spring resource spaces. During the Thule Station Period, spring subsistence strategies were aimed at filling storages with meat and blubber, which were consumed during ‘meagre’ periods of the year (mid-summer and mid-winter). The spring new ice areas off Appat (Saunders Island) in Uummannap Kangerlua (Wolstenholme Fjord) and Neqe/Pitoqafik northwest of Siorapaluk make a good example of this strategy. In early spring, many families gathered at these sites, and through communal hunting of walrus on new ice (re-formed thin ice on local ledges or open water areas), or along the fast ice edge, indispensable provision for people and their large dog teams, as well as blubber for the lamps, were secured (Freuchen and Salomonsen 1961: 135; Holtved 1967: 101). At present, accumulation of such large amounts of walrus meat does not have the same priority and is not possible due to quotas.

Past hunters, like the present, took advantage of the spring ‘resource boom’ by following the retreating fast ice edge into the fjords. This included hunting migrating narwhals and belugas along the ice edge, ringed and bearded seals on the fast ice, and seabirds in openings in the ice. The rich and diverse resource spaces along the ice edge, shear zones like ‘The Mouth of the Sea’ in Qimusseriarsuaq (Melville Bay) south and southeast of Savissivik, and the ice berg banks close to Qeqertarsuaq (Herbert Island), as seen by the tracking of the present hunting trips, are emphasized in the historical sources, as well.

#### Summer resource spaces

From the GPS tracking, we have seen that the open water season is characterized by intensive motorboat traffic between Qaanaaq and the inner parts of Kangerlussuaq (Inglefield Bredning), where the hunters board kayaks and hunt narwhal. In contrast, summer resource spaces were considerably more restricted during the Thule Station Period, when the kayak was the only sea-going vessel owned by Inughuit. This situation changed during the Post-Thule Station Period, when small cutters were purchased by some hunters. These vessels served partly as hunting vessels, partly as mother ships for 2–4 kayaks during long trips aimed at open water hunting of narwhal, and it became possible to transport large quantities of meat and blubber back from distant hunting grounds during summer. The cutters thus facilitated the formation of new resource spaces in the open water season (Gilberg 1971: 8).

Historically, the large seabird colonies of thick-billed murre (*Uria lomvia*), common eider (*Somateria mollissima*), and little auk were important summer resources. This has changed in recent times due to legal restrictions on the hunting of eiders and murres during the breeding season. People still catch little auk in areas where breeding colonies are located close to permanent settlements (Savissivik, Siorapaluk), and although some families relocate for some time each summer to catch little auk, this does not happen with the same frequency as it did in the Thule Station Period (see Mosbech et al. 2018).

In present times, Arctic char is typically caught in nets at the mouth of river deltas. During the Thule Station Period, char fishing was also conducted from summer tent camps by lakes in the inland around Kangerlussuaq (Inglefield Bredning). In this resource space, some caribou were hunted as well. Long hunting trips in late summer/autumn for caribou were sometimes made via sledge routes over the inland ice to Inglefield Land (Holtved 1967: 108). Today, fast motorboats are used to reach these distant caribou hunting grounds.

#### Autumn resource spaces

The autumn resource spaces have changed through times. In the Thule Station Period, hunting on new ice of walrus and ringed seal—for instance, around Appat (Saunders Island)—was of great importance. Meat and blubber from these hunts were indispensable as stored resources for the winter. This specialized new ice hunting ceased during the Post-Thule Station Period and the present hunters use motorboats for walrus hunting in the autumn.

We have seen from *Piniariarneq* that many of the present hunters in Kangerlussuaq (Inglefield Bredning) prioritize Greenland halibut fishing as soon as the sea ice forms over the fishing grounds. This important commercial resource was not exploited earlier. During the Post-Thule Station Period, some Greenland halibut were caught, but fish were mostly used for feeding dogs, not for export, and earlier again as emergency food. Greenland halibut fishery is a model example of how radically the emergence of a new resource, in this case, a new ‘cash crop’, can change the distribution of resource spaces.

#### Winter resource spaces

Today, winter is marked by Greenland halibut fishery through holes in the fast ice in Kangerlussuaq (Inglefield Bredning). In contrast, during the Thule Station Period, the winter season was a time when hunting of ringed seals at their breathing holes and, not the least, consumption of stored resources, formed the subsistence base.

In addition, in contrast to the present, fox fur constituted the all-dominant commodity during the Thule Station Period. Winter resource spaces for procuring foxes were situated close to settlements and caches, and sometimes, bait in the shape of whole seals was placed close to the trap lines (Ivik 1992). Trapping and preparation of fox skins for trading with the Thule Station and the stations in Savissivik and Siorapaluk formed an important part of the winter activities of the families (Holtved 1967: 108–111). In a way, the trading posts themselves emerged as resource spaces. They offered food security during late winter and of course European trade goods (Grønnow 2016: 9–10). The fox fur trade waned during the Post-Thule Station Period as the Royal Greenlandic Trade prioritized buying seal skins from the hunters due to developments in the international fur market (data from Statistisk Årbog 1953–1984). This, in turn, lead to intensified use of sealing nets, originally introduced during the Thule Station Period (Holtved 1967: 105).

Umimmattooq (Ellesmere Island) on the Canadian side of the Nares Strait was a resource space during the Thule Station Period. When sea ice conditions and supplies allowed, groups of hunters and sometimes families made long-lasting late winter expeditions across the Nares Strait to hunt muskoxen, caribou, and polar bear. However, these travelers risked forgoing hunting opportunities at home such as the important early spring communal walrus hunt. The Canadian enforcement of the eastern border during the 1950s put an end to these long hunting trips. In contrast, the prestigious polar bear hunting in Melville Bay shows continuity from the historic periods until the present. The polar bear has always played an important role, not only as a meat and clothing resource (e.g., Ivik 1992: 52), but also as a trade item and, not least, as a cultural marker of Inughuit society.

## Discussion: Resource space dynamics

Involving occupational hunters in the study of spatio-temporal patterns of resource utilization has revealed many nuances in the way that different hunters engage resources, or put in another way, where, when, how and why their resource spaces emerge. Some hunters are avid narwhal hunters and kayak paddlers, while others are not. Again, some seem especially attuned to hunting polar bear, walrus, or land mammals, while others have also taken to Greenland halibut fishing. We have quantified and mapped each of the hunters’ topographical trajectories into a seemingly unified whole, but it is worth noting that this mapping exercise may mask individual variability in how the resource spaces emerge.

This also relates to the question of representativeness. How representative are the *Piniariarneq* data of the hunting practices of occupational hunters in Avanersuaq today? Seen from one perspective, one could argue that our sample is biased in Qaanaaq’s favour, and does not adequately represent hunters from Siorapaluk and Qeqertat. Arguably, routes and resource spaces would have been distributed somewhat differently had our data included hunters from these villages as well. It can also be speculated to what extent the hunters who tracked their routes are representative of all the hunters in Qaanaaq and Savissivik. The cross section of the participating hunters is wide, ranging from individuals who are considered big hunters to others who are not. Some hunters were young and some middle aged. From that perspective, we may argue that our sample represents the diversity of hunters, and confronted with the results of our analyses in dialogue meetings, the hunters concur that many of the patterns revealed are “typical” of the overall use of the landscape.

From a qualitative perspective, the notion of representation begs the question: “what” or “who” the participating hunters should be representative of, and who sets these perimeters in the first place: the hunters, the GPS technology, or the scientists. Would a so-called representative selection of hunters appropriately account for the myriad of social variables that also plays into any given hunter’s use of resources, his preferred hunting areas, and the frequency with which he hunts, such as tenure, family history, marital status, food preferences, skill and expertise, social networks and extended families? From this perspective, the *Piniariarneq* dataset could never represent the hunters “completely”.

Had the *Piniariarneq* project spanned for longer than 12 months there is little doubt that it would have revealed how the dynamics of resource spaces are influenced by year-to-year variation. We know for a fact that the variation between years is pronounced, and several of the participating hunters expressed desire to continue the project, to demonstrate these temporal dynamics. The narwhals that suddenly appeared in Illaarsussuaq (Sidebriksfjord) in 2015 are a case in point. Evidently, the narwhals did something unusual that year, but what is important here is that we know about it today, because the hunters located the narwhals, and so, a new resource space emerged for a time.

It emerges quite clearly from the GPS data and the historical record that resource spaces are neither stable in terms of location nor in terms of time. The very concept of resource space, therefore, cannot mean the same thing, all the time. Resource spaces shift across the entire region over time: seasonally, because the seasons are marked by different resource availability at different places; and over longer time scales, since what may once have been an important resource is not important anymore (e.g., foxes), and new resources emerge (e.g., Greenland halibut). The warming of the Arctic and the resulting reduction in sea ice within the last 2 decades has also meant that resources are engaged in different ways, at different times of the year, and at different places in the landscape. During the Thule Station Period, dog sledge infrastructure was all dominant, and most resource spaces were in one way or another premised by sea ice as a hunting platform, be it new ice or fast ice. This often rendered the summer a problematic period. Today, the introduction of fast motor boats has meant that the hunters are capable of taking full advantage of the expanding open water season, and the summer narwhal hunt in the bottom of the fjords, which is the source of a very important cash income, connects with motor boat logistics. Thus, an interplay between environmental change and introduction of new technology has contributed to a significant reconfiguration of resource spaces in recent times. There are, however, also striking continuities. Hunters today continue to pursue many of the same resources that they did in historic and pre-historic time: ringed seal, narwhal, walrus, polar bear, caribou, little auk, and so forth; and although the raw materials used to fashion tools and hunting equipment have changed, some of the technology, and its usage, remains remarkably similar. Moreover, some of the old sledge routes remain in use, as do many of the old hunting areas and camps; albeit, not in the same way or with the same regularity.

The continuities and discontinuities do not cancel each other out. Instead, they speak of a dynamic, which is owed to different overlapping factors that all feed into the “eventness” of resource spaces. Sometimes the formation of a particular resource space is overwhelmingly down to a hunter’s choice, vision, opportunity, and what is often referred to as local knowledge. Like the resource spaces themselves, however, knowledge is never wholly local or global. Knowledge is dynamic and transforms, thus accounting for the fact that hunters do not engage resources, resource spaces, trails, and hunting camps in the same way in the present, or historically over time.

While it is the case that there can be no resource spaces without humans extracting the resources, it is of course also the case that there can be no resource spaces without animals. They are a defining part of the event, and animal distribution and movement patterns are, therefore, also crucial for understanding which segments in time and space become resource spaces. The narwhal hunt in the bottom of Kangerlussuaq (Inglefield Bredning) in summer is a good example of how the Qaanaaq hunters take advantage of a well-known, seasonally re-curring concentration of animals. The little auk is another case in point. It is abundant immediately adjacent to Savissivik, which explains why the GPS data reveal hunters (and people more generally) in Savissivik catch little auk in larger volumes and with greater frequency than the Qaanaaq participants, who need to travel approx. 60 km to reach the nearest little auk colony (cf. Boertmann and Mosbech 1998). The more prominent role of walrus in Qaanaaq compared to Savissivik is also in good accordance with the fact that Qaanaaq is situated relatively close to Iluleerloq (Murchison Sound), which is an important concentration area of walrus (Heide-Jørgensen et al. 2017). In contrast, fewer harp seals are caught in Qaanaaq compared to Savissivik, which no doubt partly reflects that relatively fewer harp seals reach as far north as Qaanaaq during their northbound summer migration in Baffin Bay (Rosing-Asvid and Dietz 2017). We also note that the two participating hunters from Savissivik caught a significantly larger number of polar bears than the fifteen hunters from Qaanaaq. This relates to the geographical distribution and habitat preferences of polar bears, the polar bear sub-population around Savissivik being much larger than the polar bear sub-population around Qaanaaq (SWG 2016). Savissivik is also historically known as an area with many polar bears and frequently portrayed in the literature as the home of the polar bear hunters (Rasmussen 1945). However, the influence of polar bear abundance on the relative size of the catches is mediated indirectly through quotas. Hunters in Qaanaaq and Savissivik are allocated different quota sizes (6 to Qaanaaq and 14 to Savissivik), precisely because of the different polar bear sub-population sizes, so, in this case, national wildlife management strategies are also part of the story.

We must also factor in economic opportunity. The Greenland halibut in Kangerlussuaq (Inglefield Bredning) is a relatively new and important resource to hunters in Qaanaaq, whereas it hardly constitutes a resource in Savissivik. This is not so because the Greenland halibut has suddenly arrived to Kangerlussuaq, nor is it the case that it absent in the waters near Savissivik. Rather, the difference is owed to the presence of a fish processing plant in Qaanaaq, and lack thereof in Savissivik. With no facility to receive and prepare the catch for the national and international market, Greenland halibut cannot emerge as a resource in Savissivik. In contrast to Qaanaaq, the GPS data reveal that seals remain an important resource in Savissivik, which is not only due to their marked local abundance and suitability as dog food, but also the fact that sealskin is the only item that can be traded in Savissivik. As also seen in the case of fox furs and seal skins during the Thule Station Period and the Post-Thule Station Period, respectively, the local dynamics of resources and resource spaces are, therefore, closely tied to the presence of an infrastructure for trading in the catch, and in turn also subject to national and international market trends and fluctuations.

The dynamics of resource spaces, therefore, emerge in the convergence of local phenomena, e.g., wildlife concentration areas, and global processes, such as climate change, political decisions, and economic investment that originate far beyond the resource spaces themselves. Resource spaces are also, as we have shown, deeply entwined with settlement patterns. The GPS data have revealed how the vast majority of resources are harvested within a home range of only 30–60 km from the towns. On a regional scale, this translate into an intensive use of a relatively small area, centred around towns, the locations of which are the consequences of various historical processes, including establishment of colonial trade stations and forced relocation. In contrast, the historical periods were characterized by a less intensive use of a much larger portion of the landscape, due to a settlement pattern consisting of numerous small settlements distributed across the entire region. We should note, however, that the overall practice of resource utilization today in some ways remains similar to the historical periods. Both today and in historic times, the hunters predominantly bag their resources in relative proximity to home. However, today, the range of many individual hunting trips is larger (and the time span shorter) than in historic times owing to the introduction of motor boats. What has changed then, is not so much the way in which hunters engage resource spaces, but rather the perimeters given by centralization, rendering use of distant resource concentrations increasingly infrequent and unfeasible, unless the hunting trips are focused on big game like narwhal, walrus and polar bear, caribou, or muskoxen.

## Conclusions

Drawing on *Piniariarneq* data and historical records, this article has explored the notion of resource spaces as emergent through human engagement in the present, and over time. Rather than being finite in terms of space and time, it is shown how resource spaces are dynamic events that occur through rather complex structures and interests that originate and have relevance and meaning both within and far beyond the resource spaces themselves. Our approach to studying resource spaces through the perspective of human activity is in a sense a study of human societies in movement and transition. Resource spaces change with human societies, and vice versa, in a way that speaks to the close interconnectedness between humans and their environment and bears relevance to spatial planning and the management of living resources in Greenland.

Like other parts of the Arctic, Greenland is committed to the study and protection of biodiversity in response to climate change and an anticipated industrialization of the region in the near future. Thus, at present, Greenland is the scene of mapping of key habitats for important plants and animals, biodiversity hotspots, and ecosystem functioning, the results of which feed into political spatial planning processes. Mapping of important resource spaces for local communities has also been conducted on a number of occasions, but only rarely through the approach of direct action of hunters and local communities *showing* what they do—rather than what they, or scientists, *say* they do. Electronic logs of fishing vessels and the mandatory hunting bag recording system, maintained by the Greenland Ministry of Fishing and Hunting, continue to provide important spatial information at least for larger fishing vessels and harvest of game species under quota. Through time, various interview studies, often undertaken by biologists or with a biological focus, have been used to map hunting and small-scale fishing areas, as well as local knowledge pertaining to biodiversity.^7^ In addition, long-term fieldwork carried out by anthropologists and ethnographers, both participating in and following hunters in situ, provides another layer of contextualized knowledge, which is, however, rarely quantifiable and reducible to a map, and thus not usually compatible with data on animal distribution and movement patterns collected by biologists.

It is the view of the authors that an approach like *Piniariarneq* contributes to a better integration of important human resource spaces in spatial planning processes, and not least a better rooting of knowledge production in the local communities. The method allows for collection of data on human use of the landscape, which are in many respects compatible to biological data, better facilitating integrated analyses, and assessments in a broad ecosystem based approach to management (humans as part of the ecosystem). We have presented data only for 1 year, which is not representative for the distribution and dynamics of resource spaces in Avanersuaq over a longer temporal scale. In that sense, *Piniariarneq* is a pilot project. However, implemented over more years and with more participants, and with the results subjected to dialogues in forums of hunters and supported by existing interview based/participatory study approaches, robust knowledge on human resource spaces may be attained. In this way, human resource spaces may be part an important contribution to spatial planning processes.

## Electronic supplementary material

Below is the link to the electronic supplementary material.

Supplementary material 1 (PDF 831 kb)

## Footnotes

1 For further information pertaining to the Thule Trading Station and its closure in 1953, see Petersen (1996) and Brøsted and Fægteborg (1987) respectively.

2 Unless otherwise stated, our use of the word landscape refers also to seascape and icescape (sea ice and glaciers), since many trails, trips, and hunting activities involve one or more of these disparate, yet interconnected “scapes”.

3 Qeqertat lost its official status as a “village” in the mid-eighties, and was therefore not included in Greenland Home Rule Government’s (1979–2009) implementation of services and infrastructure on par with other towns and villages throughout the country. In effect, there is no electricity in Qeqertat. In time, villagers have acquired their own generator for electricity to run a communal washing machine and water-heater in the village service house. The only telecommunication in the village is a satellite telephone, since the telecommunications mast that was erected, does not function due to the lack of electricity.

4 Local place names are spelled using West-Greenlandic spelling throughout.

5 Dog teams and motorized boats are also used for local transportation, which is not always hunting related.

6 Table shows average gross household income by district, time (2002–2015), number of adults and children. See Qaanaaq. Figures includes villages.

7 For instance, in preparation of The Environmental Oil Spill Sensitivity Atlas of the Greenland Coastal Zone, focus group interviews were conducted with hunters and fishermen to ensure that important local resource spaces were taken into account in oil spill response and contingency plans (Olsvig and Mosbech 2003).
