# Supplementary material for: Present and past dynamics of Inughuit resource spaces
Source: Ambio. 2018 Mar 8;47(Suppl 2):244–64. doi: 10.1007/s13280-018-1039-6 (PMC5963569; doi:10.1007/s13280-018-1039-6)
Supplement: Supplementary file 1 — Supplementary material 1 (PDF 831 kb) [file 13280_2018_1039_MOESM1_ESM.pdf]

Ambio

Electronic Supplementary Material

This supplementary material has not been peer reviewed.

Title: Present and past dynamics of Inughuit resource spaces

Authors: Janne Flora, Kasper Lambert Johansen, Bjarne Grønnow, Astrid Oberborbeck Andersen, Anders Mosbech

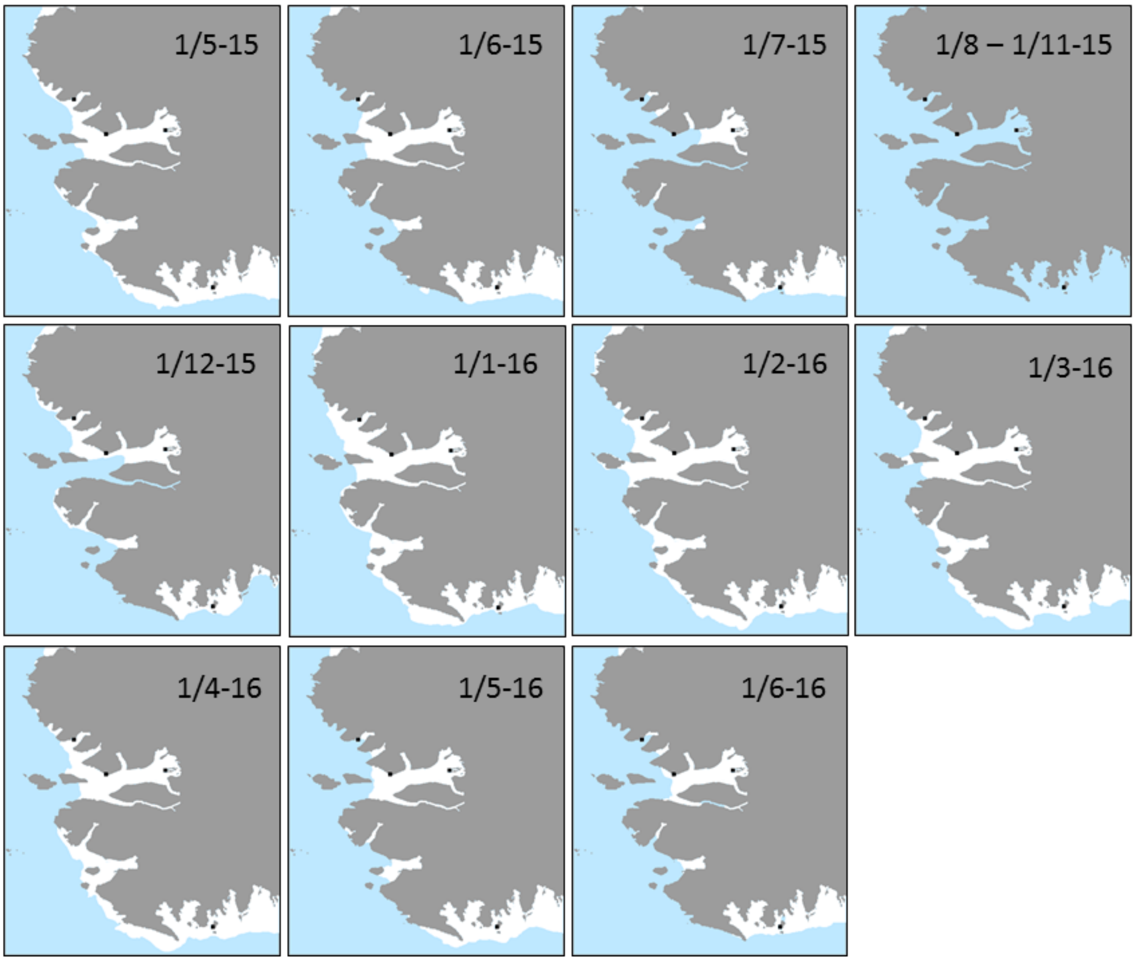

Fig. S1 The distribution of land-fast sea ice during the first week of each month over the course of the GPS tracking period, based on *Canadian Ice Service Arctic Regional Sea Ice Charts in SIGRID-3 Format, Version 1* (http://nsidc.org/data/G02171). The extent of the land-fast sea ice has decisive influence on the movement patterns of the hunters and is important to keep in mind when examining the maps of the hunters' resource spaces

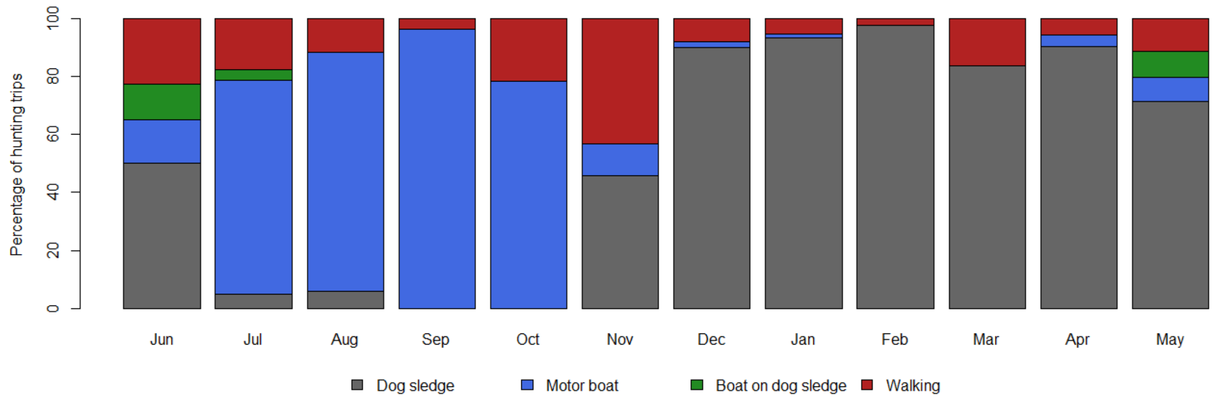

Fig. S2 Percentage of hunting trips undertaken by different means of transportation during the months of the year, based on the recordings made by hunters that participated in the *Piniariarneq* study. From November to June, the dog sledge was the dominant means of transportation, whereas the hunters almost exclusively used motor boats between July and October. Trips on foot peak in November, when there is too much new ice for motor boating, yet stabile fast ice suitable for dog sledge traffic has not yet formed

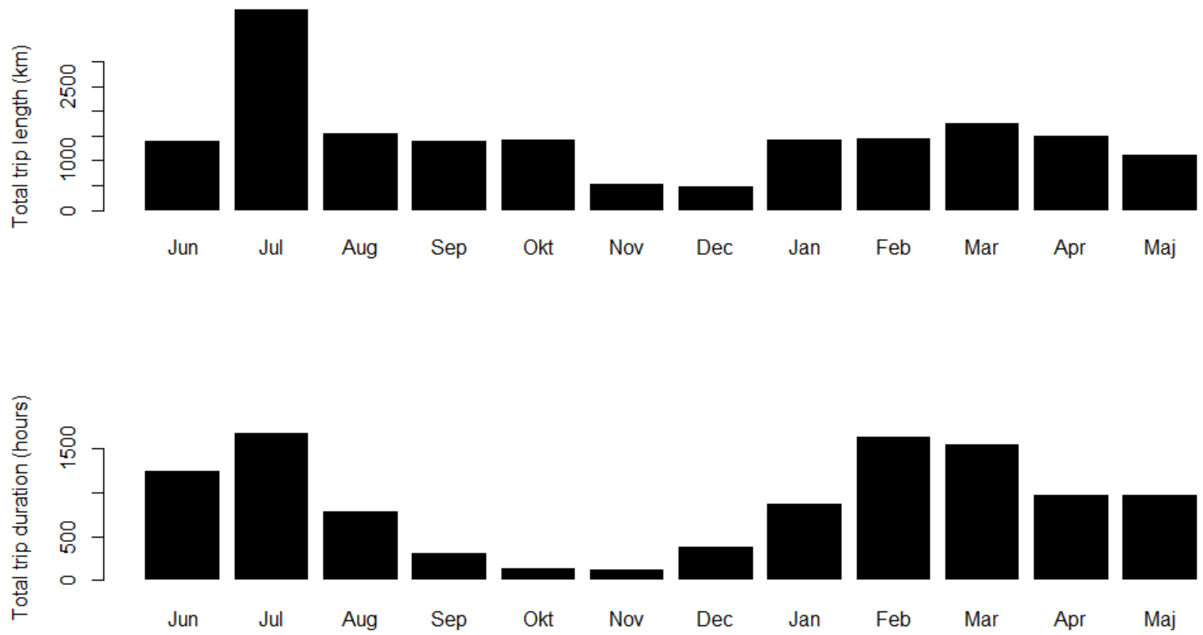

Fig. S3 Monthly total length (km) and duration (hours) of GPS tracks from hunters that participated in the *Piniariarneq* study. The raw numbers from May and June have been divided by 1.5 and 1.9 respectively to account for coverage in both 2015 and 2016 (tracking period = May 16th 2015 to June 26th 2016). There is a tendency towards a bi-modal distribution with a marked peak of mobility in July, corresponding to the beginning of the motor boat season, and another peak in February-March associated with dog sledge traffic on the land-fast ice. During the slow and gradual formation of sea ice in October-December, mobility is clearly restricted

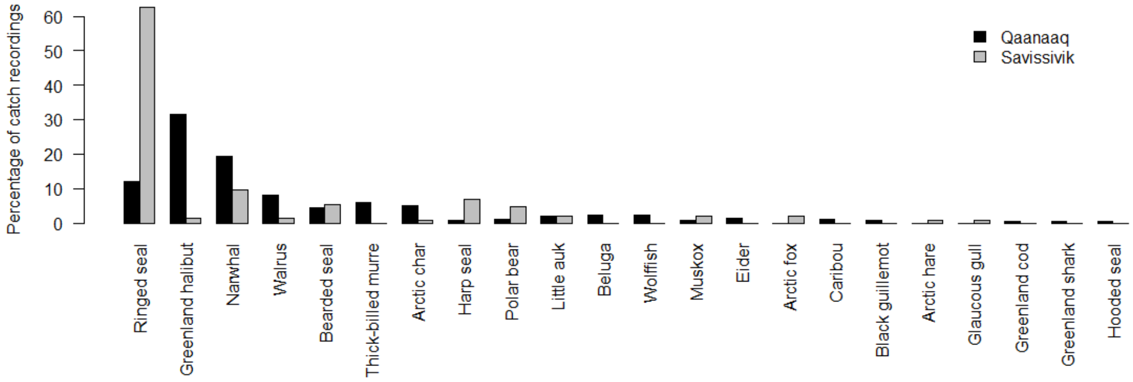

Fig. S4 Comparison between the species distribution of catches recorded by Qaanaaq and Savissivik hunters, who participated in the *Piniariarneq* study. For the comparison, the number of recorded catch events (dots on map) of each species was used, not the total number of individuals bagged (e.g. one catch event of Greenland halibut typically involves many individuals, whereas a catch event of narwhal generally does not). A few species, mainly representing by-catch from Greenland halibut fishery, were excluded from the comparison. There are a number of clear contrast between the catches of Qaanaaq and Savissivik hunters, the most prominent being the domination of ringed seal in Savissivik, and the prominent role of Greenland halibut in Qaanaaq
